# Free-Energy Calculations for Bioisosteric Modifications of A_3_ Adenosine Receptor Antagonists

**DOI:** 10.3390/ijms20143499

**Published:** 2019-07-16

**Authors:** Zuzana Jandova, Willem Jespers, Eddy Sotelo, Hugo Gutiérrez-de-Terán, Chris Oostenbrink

**Affiliations:** 1 Institute of Molecular Modeling and Simulation, University of Natural Resources and Life Sciences, 1190 Vienna, Austria; 2 Department of Cell and Molecular Biology, Uppsala University, SE-75124 Uppsala, Sweden; 3 Centro Singular de Investigación en Química Biolóxica e Materiais Moleculares (CIQUS) and Departamento de Química Orgánica, Facultade de Farmacia, Universidade de Santiago de Compostela, 15782 Santiago de Compostela, Spain

**Keywords:** Adenosine receptor, free energy calculations, molecular dynamics simulations, Groningen Molecular Simulation packace (GROMOS)

## Abstract

Adenosine receptors are a family of G protein-coupled receptors with increased attention as drug targets on different indications. We investigate the thermodynamics of ligand binding to the A_3_ adenosine receptor subtype, focusing on a recently reported series of diarylacetamidopyridine inhibitors via molecular dynamics simulations. With a combined approach of thermodynamic integration and one-step perturbation, we characterize the impact of the charge distribution in a central heteroaromatic ring on the binding affinity prediction. Standard charge distributions according to the GROMOS force field yield values in good agreement with the experimental data and previous free energy calculations. Subsequently, we examine the thermodynamics of inhibitor binding in terms of the energetic and entropic contributions. The highest entropy penalties are found for inhibitors with methoxy substituents in meta position of the aryl groups. This bulky group restricts rotation of aromatic rings attached to the pyrimidine core which leads to two distinct poses of the ligand. Our predictions support the previously proposed binding pose for the o-methoxy ligand, yielding in this case a very good correlation with the experimentally measured affinities with deviations below 4 kJ/mol.

## 1. Introduction

Adenosine is a purine nucleoside and a native chemical that is irreplaceable in the life cycle of living organisms. Adenosine fulfils a signaling function as part of cyclic adenosinemonophosphate (cAMP) and is crucial for cellular energy reservoirs as building block of adenosine triphosphate (ATP), releasing energy when converted to adenosine diphosphate (ADP). Adenosine itself accumulates in the extracellular space during metabolic stress such as ischemia, hypoxia, cell damage or inflammation [1,2] and thus serves as an alarm molecule to report tissue damage. It simultaneously triggers a series of reactions responsible for tissue protection and maintaining homeostasis [3], via interaction with one of the four adenosine receptors, A_1_, A_2A_, A_2B_ and A_3_. These receptors are G protein-coupled receptors (GPCRs), which pass on a signal via inhibition (A_1_ and A_3_) or stimulation (A_2A_ and A_2B_) of adenylyl cyclase and concomitant changes in intracellular cAMP concentrations. In this study, we focus on the chemical modulation through antagonists of the A_3_ receptor subtype (A_3_AR), which plays a crucial role in cardio protection [4], induces mast cell degranulation [5], attenuates neutrophils [6,7] and mediates a suppression of melanoma cells [8]. Inhibition of A_3_ receptors on the other hand showed protection against renal failure [9] and reduces ocular pressure.

In the last decades, different chemotypes emerged as antagonists of the A_3_AR, all of them being heterocycles of a more or less complex nature, with different chemical decorations that govern the specific structure–activity relationships (SAR) for each of them. 4-amidopyrimidines represent one of the last and most potent chemotypes with high affinity and selectivity, initially reported by the Sotelo lab [10]. The SAR within these series, which include compounds in the low nanomolar range, was explained on the basis of a binding mode that involves interactions with key residues of the adenosine A_3_ receptor. Specifically, Asn250^6.55^ (hydrogen bonding) and Phe168^EL2^ (π−π stacking) are conserved among all adenosine receptors [11,12]. The ligands bind in the classical orthosteric site common to agonists and antagonists, as opposed to other allosteric sites described for the adenosine receptors, such as the ion-binding allosteric pocket with a bound sodium ion, which is conserved among all Class A GPCRs [13]. Recently, the effect of the bioisosteric replacement of one of the nitrogens in the pyrimidine rings was reported to yield potent and selective N-(4,6-diarylpyridin-2-yl) acetamide derivatives [14]. In that work, the role of the second nitrogen of the parent 4-amidopyrimidine series was attributed to the stabilization of a water network, through extensive molecular dynamics (MD) simulations coupled to free energy perturbation (FEP) calculations.

We herein examine in detail the thermodynamics of ligand binding for the series of N-(4,6-diarylpyridin-2-yl) acetamide derivatives. We compute the effect of replacing a nitrogen atom in the pyrimidine ring by a CH group to yield a pyridine ring, depicted as Series 2 and 3, respectively, in Table 1. We examine the sensitivity of the predictions to the charge distribution in the models and compare results from different molecular dynamics (MD) simulation sampling methods and free energy protocols, i.e., the GROMOS simulation package [15] using the GROMOS 54a8 force field [16] used in this work, as opposed to the Q simulation package [17] implementing the OPLS3 force field [18,19], employed in the work of Azuaje et al. [14]. Furthermore, we compute the thermodynamic fingerprint to rationalise the differences in binding affinity in terms of energy and entropy.

## 2. Results and Discussion

**Figure 1:**
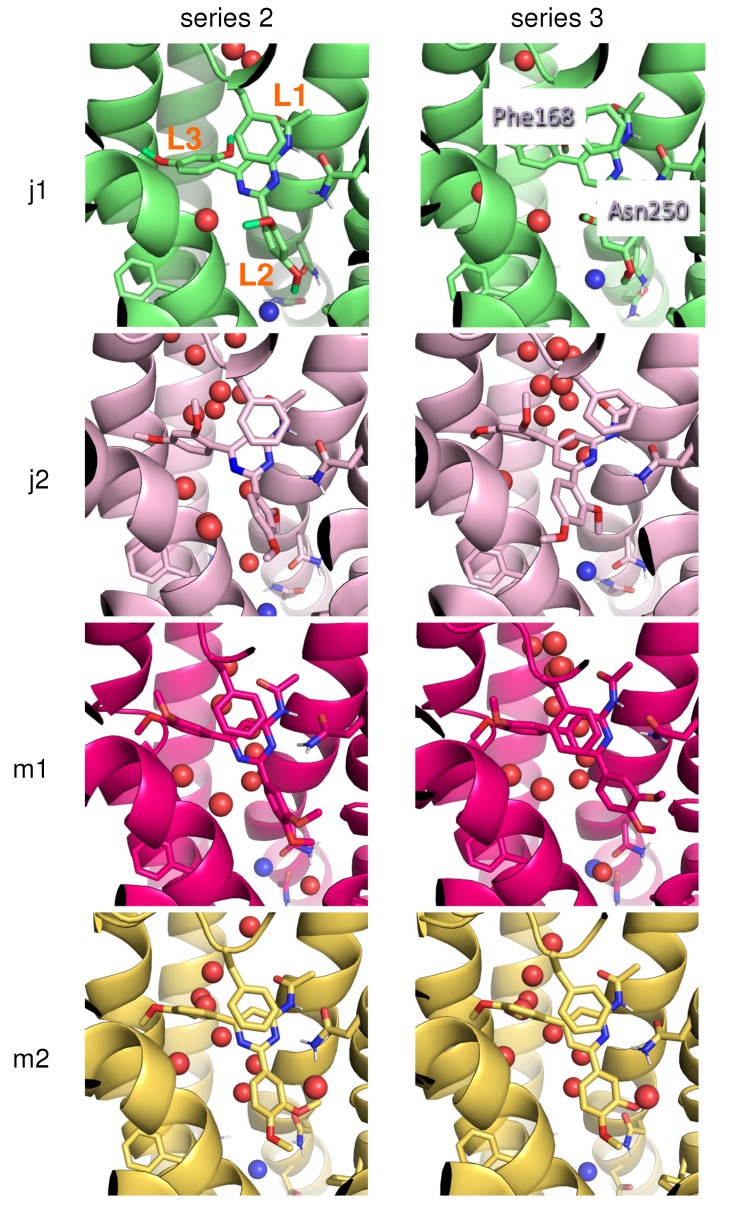
Initial and final poses of ligands in the 2 > 3 transformation for compounds j and m, in two distinct starting poses. For 2j1 the three ligands sites (L1, L2, L3) are indicated, for 2j2 the most important residues are labeled. Red spheres correspond to water molecules, the blue sphere to the Na^+^ ion.

The 3D structure of the A_3_AR was built by homology modelling from the crystal structure of A_2A_AR in complex with an antagonist, which shares 35% sequence identity with the A_3_AR [20]. The initial pose of the ligand was taken from Azuaje et al. [14], where the acetamido group (L1) of the ligand interacts with Asn250^6.55^, L2 is located in the inner cavity of the receptor, while L3 is oriented towards the more spacious extracellular direction (groups L1, L2 and L3 are indicated for compound **2j** in pose 1 in Figure 1). The complex was equilibrated for four nanoseconds of free MD under spherical boundary conditions, after which the free-energy calculations were performed.

The free-energy changes between series **2** (pyrimidine) and series **3** (pyridine) were calculated using thermodynamic integration to obtain relative binding free energies (see methods section). This comparison was performed independently for each of the decorated ligands shown in Table 1. For the cases with substituents in ortho- and meta- positions (compounds **j** and **m**), the simulations considered two alternative binding modes, which essentially differed in the rotamer of the aryl ring in the deeper L2, placing the corresponding ortho (compound **j**) and meta (compound **m**) substituents in different cavities, see the left-hand side panels of Figure 1 and ref [14]. Table 2 summarises the free-energy differences upon nitrogen substitution on the central ring. Statistical error estimates on the TI data are as low as 1 kJ/mol. To estimate the hysteresis of our calculations, we calculated the backward free-energy change for 3 g > 2 g, which lead to a value of −4.1 ± 1.9 kJ/mol, which is comparable to the forward pathway 5.6 ± 1.6 kJ/mol, leading to a hysteresis value as low as 1.5 kJ/mol.

The experimental data in Table 2 show that in every case except for the **2d** compound, removing the second nitrogen leads to decrease in affinity. Our calculations show the same general trend, although the experimental affinity increase observed for the **3d** compound (as compared to **2d**) is not captured, and we observe a small unfavourable free-energy change instead (+1.8 kJ/mol). The ranking in affinity shift for this bioisosteric replacement is correctly predicted and agrees with the predicted ranking from Azuaje et al.

**Figure 2:**
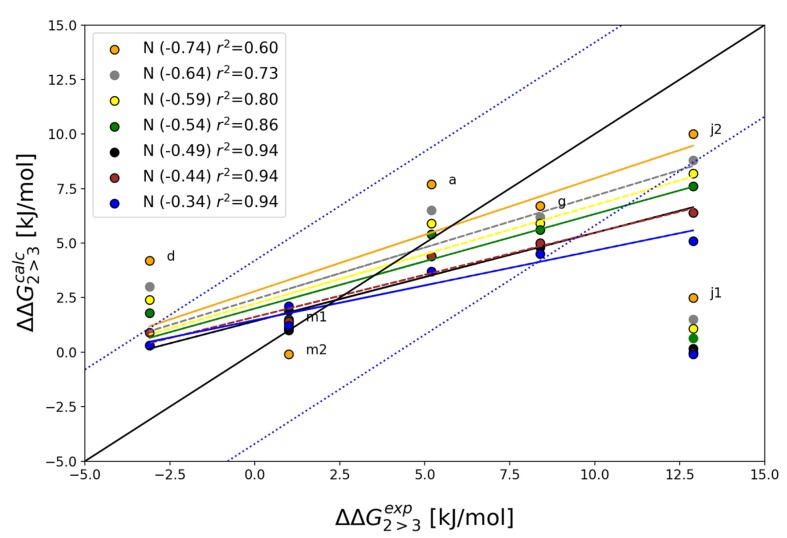
Comparison of experimental vs calculated relative binding affinities between the **2** and **3**. The black diagonal shows perfect agreement between experiment and calculations, the blue dotted lines indicate bounds to ± 4.2 kJ/mol (1 kcal/mol). Regression lines are given for the data set excluding j1.

Since the ligand atoms were not positionally restrained during the simulations, we can observe rotation of the L3 ring, but not of the L2 ring of compound **j**, which is located in a relatively narrow pocket with less conformational freedom. Both L2 and L3 rings in the **m** compounds, on the other hand, rotate more frequently during the simulation (Figure S2 in the supplementary material). As observed in Azuaje et al., the relative binding free energy calculated for pose 1 of compound **j** does not agree with the experimental data. In agreement with the frequent rotations of the L3 ring of compound **m**, the relative binding free energies for starting poses 1 and 2 are very similar. Selecting pose 2 for compound **j**, leads to a very good overall agreement between the calculated values and experiment, with a correlation coefficient of 0.86, see the green curve in Figure 2.

A critical point in bioisosteric replacement is to correctly parameterize the atoms involved. While the van der Waals parameters of aromatic carbon and nitrogen in a heterocycle are well established, there are many ways to model the partial charge distribution of a heterocycle. We investigated the sensitivity of the calculations on the partial charge value assigned to the on variable nitrogen atom between the pyrimidine and pyridine scaffolds. The end-states of the TI calculations were prolonged to 5 ns each, and the one-step perturbation method was used to predict the relative binding free energies for different charge distributions (see methods section). The default ligand parameterization according to the GROMOS 54a8 force field led to a partial charge on the second nitrogen in the pyrimidine ring of −0.54 *e*. Figure 2 shows the effect of a systematic variation of the charge distribution of this ring in the correlation with the experimental values, with the corresponding values collected in Table 3. While the correlation coefficients are slightly better for the models using smaller nitrogen charges (i.e., −0.49 *e*, −0.44 *e* or −0.34 *e*), the slopes of these correlations become less pronounced. The mean absolute error with respect to the experimental data for compounds **a**, **d**, **g** and **m** is best for nitrogen charges of −0.54 *e* or −0.49 *e*, while the deviation from the experimentally determined lower bound for compounds **j** is smaller for the larger nitrogen charge. Higher nitrogen charges (−0.59 *e*, −0.64 *e*, −0.74 *e*) yield values for compounds **j** (in pose 2) that are closer to the experimental lower bound, but show smaller overall correlation coefficients.

The final analysis focuses on the energetic and entropic contributions to the relative binding free energies. Ligand-surrounding energy differences were calculated from the prolonged end-states simulations and entropy from the difference between the free-energy change during the ligand perturbation (using a nitrogen charge of −0.54 *e*) and the energetic contributions [21]. The results are summarised in Table 4. We considered only the ligand surrounding energy and entropy, because it can be shown that the surrounding-surrounding energetic and entropic contributions cancel exactly [21,22,23]. Not surprisingly, the ligands with the bulkiest substituents, **d, j** and **m** show the largest entropy decrease upon perturbation. This effect might be explained by disturbance of the water network in the ligand surrounding in the pyridine series, since there the hydrogen bond between the variable nitrogen and a water molecule is disrupted and the larger moieties complicate water reorganisation. Conversely, for the compounds with smaller substituents on L2 compounds (**a** and **g**), a gain of entropy is observed, which we attribute to the release in these systems of the restricted water molecule in the absence of the second nitrogen. The important effect of destabilization of unfavourably positioned water molecules on ligand binding free energies and ligand resident time, is acknowledged in the field and was previously illustrated in the characterization of A_2A_ antagonists [24]. Restricted conformational freedom of protein sidechains or ligand moieties might also effect entropy contributions [25]. The most negative energy change is seen in the **2j1 > 3j1** transformation, which compensates for its high entropy loss.

## 3. Materials and Methods

**Figure 3:**
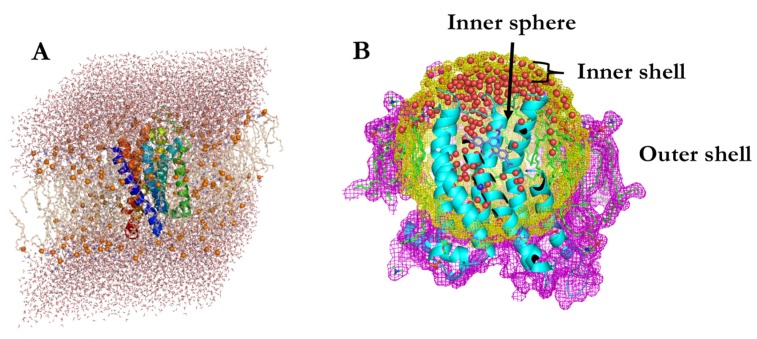
(**A**) The initial system in a triclinic box with 1-palmitoyl-2-oleoyl-sn-glycero-3- phosphocholine (POPC) membrane and water. (**B**) The same system under spherical boundary conditions. Outer shell is in magenta (atoms > 2.7 nm from the centre of geometry (COG) of the ligand), inner shell in yellow (atoms 2.2–2.7 nm from COG ligand) and the inner sphere without surface representation (atoms < 2.2 nm from COG ligand). Ligand is in light purple, the sodium ion in blue, water oxygens in red.

The starting structure of the A_3_ receptor was homology modelled based on the inactive conformation of an A_2A_ template (PDB code: 3EML) [26] as described in [10,14]. The sequence identity of the transmembrane regions amounts to 52%. It was previously used successfully to explain the structure–activity relations of a large number of compounds [10] and in free-energy calculations [14]. Ligand binding poses were also taken from the initial pose of **2g** in ref [14]. The receptor-ligand complex was subsequently inserted into a 1-palmitoyl-2-oleoyl-sn-glycero-3- phosphocholine (POPC) bilayer, with the lipid parameters taken from ref. [27]. The POPC bilayer was edited in Pymol [28] to fill x and y dimensions of a triclinic box with an edge length of 8.66 nm and edge angles 90, 90 and 60 degrees. The protein embedded in the membrane was energy-minimized in vacuum using the steepest-descent algorithm and subsequently solvated in a triclinic, periodic and pre-equilibrated box of simple point charge (SPC) water [29] with height of 10 nm (Figure 3). Water molecules automatically placed between the lipids of the bilayer or inside of the receptor helices were manually removed. This led to a system of 47,886 atoms, consisting of 166 POPC molecules, one sodium ion in the ion binding pocket, a ligand and 14 chloride counter ions. To avoid unwanted displacement of the sodium ion, it was distance restrained to the centre of mass of Trp243^6.48^, Asp58^2.50^, Ser97^3.39^ and Ser275^7.46^ with a force constant of 500 kJ mol^−1^ nm^−2^. Another minimization in water was performed using the steepest descent algorithm. All MD simulations were carried out using the GROMOS11 software simulation package [15], employing the 54a8 forcefield [30].

For the equilibration, the following protocol was used: initial velocities were randomly assigned according to a Maxwell–Boltzmann distribution at 60 K. All solute atoms were positionally restrained with a harmonic potential using a force constant of 2.5 × 10^4^ kJ mol^−1^ nm^−2^. In each of the four subsequent 20 ps MD simulations, the force constant of the positional restraints was reduced by one order of magnitude and the temperature was increased by 60 K. Subsequently, the positional restraints were removed and rototranslational constraints were introduced on all solute atoms [31]. The last two steps of equilibration were performed at 300 K, first for 100 ps under harmonic positional restraints with force constant of 15 kJ mol^−1^ nm^−2^ and afterwards under constant pressure of 1 atm for 300 ps. Anisotropic pressure scaling and a grid-based pairlist algorithm [32] were used. After equilibration, a production run of 2 ns was performed with constant number of particles, constant temperature (300 K) and constant pressure (1 atm). To sustain a constant temperature, we used the weak-coupling thermostat [33] with a coupling time of 0.1 ps. The pressure was maintained using a weak coupling barostat with a coupling time of 0.5 ps and an isothermal compressibility of 4.575 × 10^–4^ kJ^−1^·mol·nm^−3^. Solute and solvent were coupled to separate temperature baths. Implementation of the SHAKE algorithm [34] to constrain bond lengths of solute and solvent to their optimal values allowed for a 2-fs time-step. Nonbonded interactions were calculated using a triple range scheme. Interactions within a short-range cutoff of 0.8 nm were calculated at every time step from a pair list that was updated every fifth step. At these points, interactions between 0.8 and 1.4 nm were also calculated explicitly and kept constant between updates. A reaction field [35] contribution was added to the electrostatic interactions and forces to account for a homogenous medium outside the long-range cutoff using a relative dielectric constant of 61, as appropriate for the SPC water model [36]. Coordinate and energy trajectories were stored every 0.5 ps for subsequent analysis.

For simulations of ligand in water, the ligand was solvated in a rectangular box of SPC water with a minimum solute to box-wall distance of 1 nm. The equilibration and production runs were performed under conditions as described above.

### 3.1. Spherical Boundary Conditions

To save computational time, the free-energy calculations were performed under spherical boundary conditions. This can be done without a significant loss of accuracy, as long as the protein is not expected to undergo large conformational changes. After 2 ns of production run in a triclinic box, a sphere with a radius of 2.7 nm around the centre of geometry (COG) of the ligand was created. Any water molecule, ion or phospholipid that did not contain any atom within the sphere was removed (see Figure 3). Subsequently, the system was divided into three parts: the outer shell, consisting of phospholipid and protein atoms, which were outside of the 2.7 nm sphere, the inner shell, containing all atoms at a distance 2.2–2.7 nm from the COG of the ligand; water, phospholipid and protein atoms and the inner sphere, i.e., the sphere with radius smaller than 2.2 nm. Atoms in both shells were positionally restrained, the outer shell with a force constant of 83.72 kJ mol^−1^ nm^−2^ (20 kcal mol^−1^ nm^−2^), the inner shell with a force constant of 8.37 kJ mol^−1^ nm^−2^ (2 kcal mol^−1^ nm^−2^). The bond lengths in the outer shells were not constrained while bond lengths in the inner shell and the inner sphere were treated with the SHAKE algorithm [34]. The time step used in the spherical boundary conditions simulations was 1 fs, in order to avoid high particle velocities leading to failures of the SHAKE algorithm between the layers. No pressure scaling was applied and a standard, double-loop pairlist algorithm was used for calculation of nonbonded interactions. This newly created system consisted of 8160 atoms, which corresponds to a reduction of a factor 6 compared to the triclinic system. After 4 ns of simulation time of this system, the structures were taken for further free-energy calculations. In order to generate initial poses for other ligands from the **2** series, a steepest decent minimization was performed, followed by 300 ps of equilibration with newly assigned velocities at 300 K.

### 3.2. Free-Energy Calculations

The structures of all compounds in series **2**, bound to the receptor, were used as a starting pose for all of the free-energy simulations. Thermodynamic integration (TI) was used to compute the free-energy differences between ligands [37,38]. In this approach, Hamiltonian is written as a function of a coupling parameter λ, such that at λ = 0, the Hamiltonian corresponds to a compound of series 2 (state A) and at λ = 1, it describes a compound of series 3 (state B). At intermediate values, the Hamiltonian corresponds to an unphysical intermediate state. Simulations are performed at eleven discrete, equidistant λ-values and the derivative of the Hamiltonian with respect to λ are monitored. By integrating over these derivatives, we obtain the free-energy difference ($\Delta G_{A>B}$), as:

(1) $$\Delta G_{A>B}=\overset{1}{\underset{0}{\int }}{\langle \frac{\partial H\left(\lambda \right)}{\partial \lambda }\rangle }_{\lambda }d\lambda$$

At each λ value, 20 ps of equilibration were followed by 1 ns of production run. If necessary, the simulations at individual λ points were prolonged or additional λ points were added to decrease the overall error estimate below 1 kJ/mol. The end states were as well prolonged to 5 ns, in order to obtain the energy differences and estimates for modified charge distributions (see below). For perturbed atoms we used soft-core parameters of 0.5 for the van der Waals and 0.5 nm^2^ for electrostatic interactions [39]. TI was performed on the bound ligands yielding the free-energy differences in the bound state ($\Delta G_{A>B}^{b}$) and for the ligands in water, yielding the free-energy differences in the unbound state ($\Delta G_{A>B}^{u}$).

**Figure 4:**
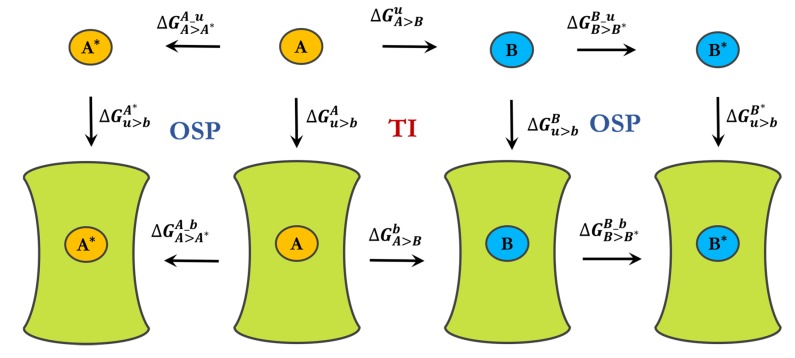
Thermodynamic cycle connecting various states. A and B represent different ligands, A* and B* ligands with modified partial charges. Δ*G_u_*_>*b*_ is the free energy of binding (u for unbound, b for bound), while Δ*G_A_*_>*B*_ is the free energy difference between states A and B. Free energy differences along horizontal arrows in the central square are computed using thermodynamic integration (TI), horizontal arrows in the flanking squares by the one step perturbation (OSP).

To investigate the influence of the charge distribution in the pyrimidine core of the molecule on the relative binding free energies we used the One-step-perturbation approach. One step perturbation is based on the Zwanzig equation [40]:

(2) $$\Delta G_{A>A^{*}}^{OSP}=G_{A^{*}}-G_{A}=-k_{B}Tln\langle e^{-\left({ℋ}_{A^{*}}-{ℋ}_{A}\right)/k_{B}T}\rangle_{A}$$

where $\langle \;\rangle_{A}$ is the ensemble average of the initial state A, $k_{B}$ is the Boltzmann constant and T is the absolute temperature. We used the prolonged simulations of the end-states A and B to calculate the free-energy changes between the original charge A used in the simulations and a modified charge $A^{*}$. This enabled us to compare free-energy differences between ligands with various charge distributions on the central ring ($G_{A^{*}>B^{*}}^{}$). In order to create a thermodynamic cycle we simulated the ligand perturbation in water ($\Delta G_{A>B}^{u}$) and in protein ($\Delta G_{A>B}^{b}$). The full thermodynamic cycle for the free-energy calculations is shown in Figure 4.

## 4. Conclusions

In this work, we provide an exploration of the effect of the bioisosteric replacement of pyrimidine by pyridine on ligand binding affinities to the A_3_ receptor. The relative binding free energies between a pyrimidine and pyridine scaffolds were computed for a series consisting on five recently reported antagonists, bearing different decorations in the symmetric aryl substituents [14]. The sensitivity of the predictions to the charge model of the pyrimidine core was examined, concluding that the default GROMOS 54a8 provides a good estimation. We also show that the examination of the binding affinities with free energy calculation methods is also a useful strategy to select the most likely binding orientation between different alternatives, as we illustrate here in the case of compounds **j** and **m**. While in the former case the ortho-methoxy substituent on the ring is specifically accommodated in one of the two possible binding orientations, in agreement with previous calculations [14], we also observe that the analogous substitution in the meta-position is equally tolerated in both orientations, since it is further away from the site of bioisosteric replacement and does not affect interactions with the key residue Asn253^6.55^. Furthermore, our current calculations provide insight into the binding thermodynamics of this series. Thus, the effect of the bioisosteric replacement on the (de)stabilization of a water network characteristic of the adenosine receptors is confirmed by the negative entropic contribution of the bulkier compounds (**d, j** and **m**), while this phenomenon is reverted if the water is not trapped in the buried area (compounds **a** and **g**). Our approach thus reveals a helpful tool in the optimization of relative binding affinities in antagonists of the A_3_ and other GPCRs.

## Figures and Tables

**Table 1:** Ligand Structures with Annotations ^a^.

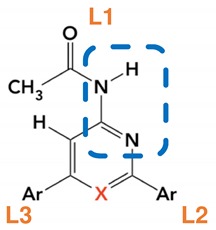	**Ligand Annotation**	**Ar**
	**a**	Ph
	**d**	4-MeCO-Ph
	**g**	4-MeO-Ph
	**j**	2,4-MeO-Ph
	**m**	3,4-MeO-Ph

^a^ The blue rectangle shows the part of the ligand that forms hydrogen bonds to Asn250. The three points of chemical decoration of this scaffold are depicted as L in orange. In this work, L1 is always an acetamide group while L2 and L3 are symmetrically substituted by the indicated aromatic groups. X indicates the point of bioisosteric replacement (N/CH).

**Table 2:** Relative Binding Free Energies between the 2 and 3 Series.

Alchemical Change	ΔΔ*G_u_*_>*b*_ (calc)^a^ [kJ/mol]	ΔΔ*G_u_*_>*b*_ (exp) [kJ/mol]	ΔΔ*G_u_*_>*b*_ (calc)^b^ [kJ/mol]
2a→3a	5.4	5.2	7.7
2d→3d	1.8	−3.1	
2g→3g	5.6	8.4	13.3
2j1 → 3j1	0.6	>12.9	−6.5^c^
2j2→3j2	7.6	>12.9	26.4^d^
2m1→3m1	1.1	1	
2m2→3m2	1.5	1	

^a^ Values calculated in this work; ^b^ values from ref. [14]. ^c^ the value corresponds to the average of poses a and d in ref [14]. ^d^ the value corresponds to the average of poses b and c in ref [14]. See Figure S1 in the supporting information for an overlay of the poses observed in this work and in ref [14].

**Table 3:** Relative Binding Free Energies in kJ/mol between the 2 and 3 Series for Different Partial Charges on N1.

Alchemical Change	N1 Charge						
	−0.74	−0.64	−0.59	−0.54^a^	−0.49	−0.44	−0.34
2a→3a	7.7	6.5	5.9	5.4	4.4	4.4	3.7
2d→3d	4.2	3	2.4	1.8	0.3	0.9	0.3
2g→3g	6.7	6.2	5.9	5.6	4.8	5.0	4.5
2j1 → 3j1	2.5	1.5	1.1	0.6	0.2	0.0	−0.1
2j2→3j2	10.0	8.8	8.2	7.6	6.4	6.4	5.1
2m1→3m1	1.4	1.1	1.1	1.1	1.0	1.4	2.1
2m2→3m2	−0.1	1	1.3	1.5	1.9	1.4	1.2
MAE^b^	3.0	2.4	2.2	2.1	2.1	2.2	2.4
r^2 c^	0.60	0.73	0.80	0.86	0.94	0.94	0.94

^a^ Charge used in the simulations. ^b^ Mean absolute error (MAE) is calculated between the calculated and experimental values for compounds **a**, **d**, **g**, and **m**; where for compound m the average between poses **m1** and **m2** is taken. ^c^ r^2^ Correlation coefficient (values are calculated without the j1 pose).

**Table 4:** Energetic and Entropic Contributions of Different Ligand Transformations in kJ/mol.

Alchemical Change	Ligand in Water			Ligand in Protein			Ligand in Protein - Ligand in Water		
	∆E	σ_Δ__E_^a^	T∆S	∆E	σ_Δ__E_^a^	T∆S	∆∆E	σ_Δ__E_^a^	T∆∆S
2a→3a	−88.9	0.5	−6.1	−78.7	2.0	−1.4	10.1	2.0	4.8
2d→3d	−88.6	0.5	−6.0	−90.3	3.3	−9.5	−1.7	3.3	−3.5
2g→3g	−88.5	0.5	−5.6	−79.4	1.2	−2.1	9.1	1.3	3.5
2j1 → 3j1	9.8	0.7	1.3	0.5	2.3	−8.6	−9.3	2.4	−9.9
2j2→3j2	8.0	0.6	−0.3	7.9	1.7	−8.0	−0.1	1.8	−7.7
2m1→3m1	−93.2	0.8	−8.7	−93.5	5.4	−10.0	−0.3	5.5	−1.4
2m2→3m2	−91.6	0.8	−7.2	−94.5	1.3	−11.7	−2.9	1.5	−4.4

^a^ Error estimate on the energy change from block averaging.

## Supplementary Materials

Supplementary materials can be found at https://www.mdpi.com/1422-0067/20/14/3499/s1.

## Abbreviations

A_3_AR	A_3_ Adenosine receptor
FEP	Free energy perturbation
GPCR	G protein-coupled receptor
MD	Molecular dynamics
POPC	1-palmitoyl-2-oleoyl-sn-glycero-3-phosphocholine
SAR	Structure-activity relationships
TI	Thermodynamic integration
