# Supplementary material for: Free-Energy Calculations for Bioisosteric Modifications of A3 Adenosine Receptor Antagonists
Source: Int J Mol Sci. 2019 Jul 16;20(14):3499. doi: 10.3390/ijms20143499 (PMC6679372; doi:10.3390/ijms20143499)
Supplement: Supplementary file 1 [file ijms-20-03499-s001.pdf]

Supplementary material

Free-energy calculations on potential A_3 adenosine receptors inhibitors

Zuzana Jandova,[1] Willem Jespers,[2] Eddy Sotelo,[3] Hugo Gutiérrez-de-Terán,[2] Chris Oostenbrink[1]

1 Institute of Molecular Modeling and Simulation, University of Natural Resources and Life Sciences,Vienna

2 Department of Cell and Molecular Biology, Uppsala University, Uppsala SE-75124

3 Centro Singular de Investigacioń en Química Biolox́ica e Materiais Moleculares (CIQUS) and Departamento de Química Orgańica, Facultade de Farmacia, Universidade de Santiago de Compostela, 15782, Santiago de Compostela, Spain

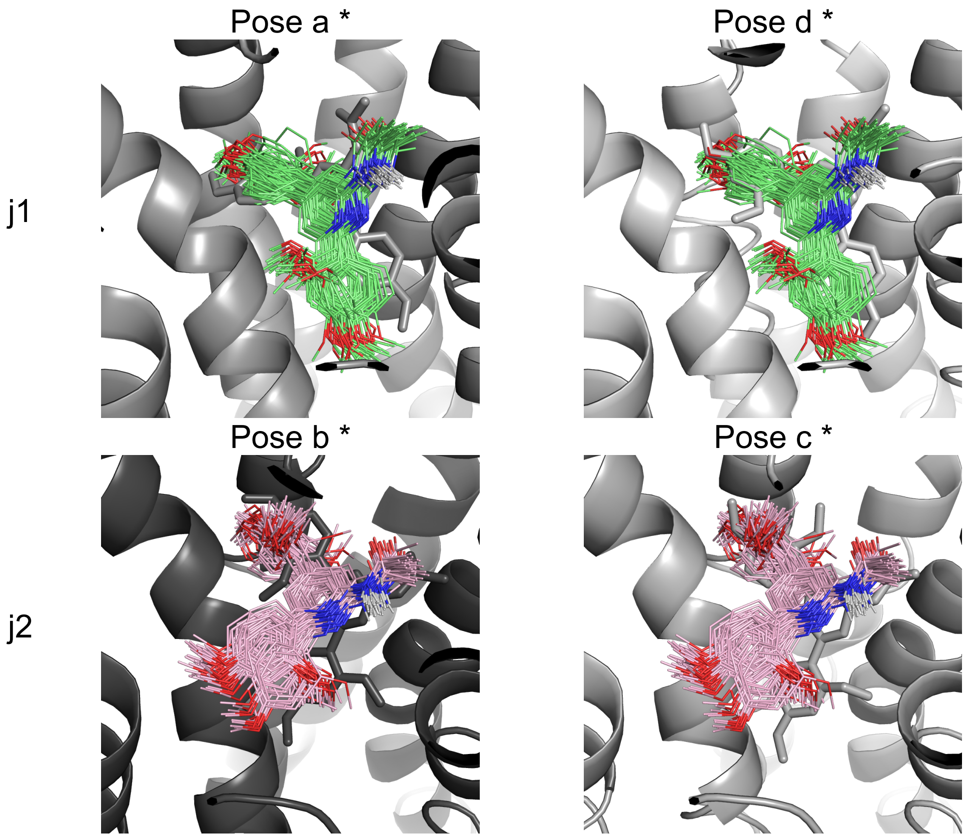

Figure S1: Overlay of trajectory snapshots of 2j1>3j1 and 2j2>3j2 with ligand in lines with poses from Azuaje et al. in grey sticks.

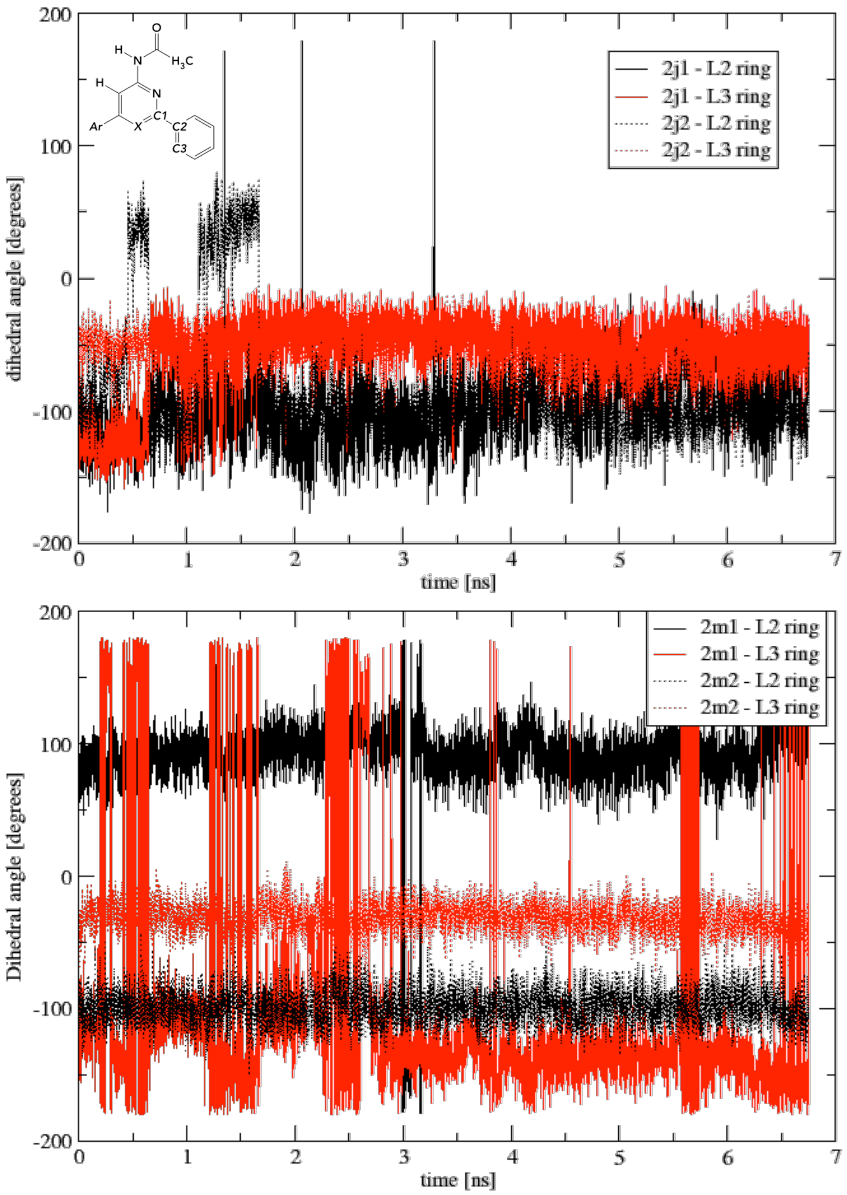

Figure S2: Time series of dihedral angles between X-C1-C2-C3 on both L2 and L3 rings for **m** and **j** compounds in starting poses 1 and 2.
